# Retraction Note: SHCBP1 promotes synovial sarcoma cell metastasis via targeting TGF-β1/Smad signaling pathway and is associated with poor prognosis

**DOI:** 10.1186/s13046-026-03774-8

**Published:** 2026-07-15

**Authors:** Changliang Peng, Hui Zhao, Yan Song, Wei Chen, Xiaoying Wang, Xiaoli Liu, Cheng Zhang, Jie Zhao, Ji Li, Guanghui Cheng, Dongjin Wu, Chunzheng Gao, Xiuwen Wang

**Affiliations:** 1 Department of Orthopaedics, The Second Hospital of Shandong UniversityShandong University, Jinan, China; 2 Department of Orthopedics, Beijing Chaoyang Hospital, Capital Medical University, Beijing, China; 3 Department of Nephrology, The Second Hospital of Shandong UniversityShandong University, Jinan, China; 4 Beijing Institute of Pharmacology and Toxicology, Beijing, China; 5 Department of Pathology, The Second Hospital of Shandong University, Shandong University, Jinan, China; 6 Department of Hematology, The Second Hospital of Shandong University, Shandong University, Jinan, China; 7 Central Research Laboratory, The Second Hospital of Shandong University, Shandong University, Jinan, China

**Retraction Note: J Exp Clin Cancer Res 36, 141 (2017)**

**https://doi.org/10.1186/s13046-017-0616-z**

The Editor-in-Chief has retracted this article because concerns were raised regarding the following figures:

- Fig. 3a SW982, 0h, untreated appears highly similar to Fig. 3a SW982, 0h LV-Control
- Fig. 3b LV-Sh-SHCBP1 Migration appears highly similar to Fig. 6c LV-Control HS-SY II
- Fig. 6b SHCBP1 (SW982) appears highly similar to Fig. 6b (cyclin D1, KYSE 30: vector and SOX9; KYSE140 vector) of [1]
- Fig. 6b E-Cadherin (SW982) appears highly similar to Fig. 6b (p21, KYSE30: vector and SOX9; KYSE140 vector) of [1]
- Fig. 5a, vimentin, HS-SY-II appears highly similar to Fig. 6D (P 27) of [2]
- Fig. 6a TGF-B1, SHCBP1, SW982 appears highly similar to Fig 5c (MMP2) of [3]
- Fig. 7a pSmad 2, SW982 appears highly similar to Fig. 5e cdc42 of [4]

The Editor-in-Chief therefore no longer has confidence in the presented data.

X Wang agrees with this retraction. C Peng, H Zhao, Y Song, W Chen, X Wang, X Liu, C Zhang, J Zhao, J Li, G Cheng, D Wu and C Gao did not respond to correspondence from the Publisher about this retraction.
